# Paediatric eye and vision research participation experiences: a systematic review

**DOI:** 10.1186/s13063-022-07021-1

**Published:** 2023-01-28

**Authors:** Jacqueline Miller, Katherine Curtis-Tyler, Michelle Maden, Annegret Dahlmann-Noor, Jane Chudleigh

**Affiliations:** 1 King’s College London & Moorfields Eye Hospital NHS Foundation Trust, London, UK; 2 University of London, London, UK; 3 University of Liverpool, Liverpool, UK; 4 NIHR Biomedical Research Centre at Moorfields Eye Hospital NHS Foundation Trust, and UCL Institute of Ophthalmology, London, UK; 5 King’s College London, London, UK

**Keywords:** Research participation, Research experiences, Children, Young people, Ophthalmology, Eye, Vision

## Abstract

**Background:**

For children and young people with eye and vision conditions, research is essential to advancing evidence-based recommendations in diagnosis, prevention, treatments and cures. Patient ‘experience’ reflects a key measure of quality in health care (Department of Health. High Quality Care for All: NHS Next Stage Review Final Report: The Stationery Office (2008)); research participant ‘experiences’ are equally important. Therefore, in order to achieve child-centred, high-quality paediatric ophthalmic research, we need to understand participation experiences. We conducted a systematic review of existing literature; our primary outcome was to understand what children and young people, parents and research staff perceive to support or hinder positive paediatric eye and vision research experiences. Our secondary outcomes explored whether any adverse or positive *effects* were *perceived* to be related to participation experiences, and if any interventions to improve paediatric ophthalmic research experiences had previously been developed or used.

**Methods:**

We searched (from inception to November 2018, updated July 2020) in MEDLINE, Embase, CINAHL, Web of Science, NICE evidence and The Cochrane Library (CDSR and CENTRAL), key journals (by hand), grey literature databases and Google Scholar; looking for evidence from the perspectives of children, young people, parents and staff with experience of paediatric ophthalmic research. The National Institute for Health Research (NIHR) Participant in Research Experience Survey (PRES) (National Institute for Health Research. Research Participant Experience Survey Report 2018–19 (2019); National Institute for Health Research. Optimising the Participant in Research Experience Checklist (2019)) identified ‘five domains’ pivotal to shaping positive research experiences; we used these domains as an ‘a priori’ framework to conduct a ‘best fit’ synthesis (Carroll et al., BMC Med Res Methodol. 11:29, 2011; Carroll et al., BMC Med Res Methodol. 13:37, 2013).

**Results:**

Our search yielded 13,020 papers; two studies were eligible. These evaluated research experiences from the perspectives of parents and staff; the perspectives of children and young people themselves were not collected. No studies were identified addressing our secondary objectives. Synthesis confirmed the experiences of parents were shaped by staff characteristics, information provision, trial organisation and personal motivations, concurring with the ‘PRES domains’ (National Institute for Health Research. Optimising the Participant in Research Experience Checklist (2019)) and generating additional dimensions to participation motivations and the physical and emotional costs of study organisation.

**Conclusions:**

The evidence base is limited and importantly omits the voices of children and young people. Further research, involving children and young people, is necessary to better understand the research experiences of this population, and so inform quality improvements for paediatric ophthalmic research care and outcomes.

**Trial registration:**

Review registered with PROSPERO, International prospective register of systematic reviews: CRD42018117984. Registered on 11 December 2018.

**Supplementary Information:**

The online version contains supplementary material available at 10.1186/s13063-022-07021-1.

## Background

Clinical research will improve the healthcare we are able to deliver [1]. Ethically robust paediatric clinical studies work in partnership with children, young people and their families to weigh concerns about childhood vulnerability and their complex care needs, with the need for children’s participation [2–5]. Paediatric research design needs to be feasible and acceptable to children and to their families [6]. Caldwell et al. [4] highlight the perils of “piggy-backing” children and young people (CYP) onto a research design intended for adult participants, where their child-specific clinical outcomes, needs and priorities may be neglected. Gillies et al. [7] discuss the ethical risks of poor experiences beyond trial entry (generally, not specifically in paediatrics), which can jeopardise retention and compromise the robustness of results. Patient ‘experience’ alongside safety and effectiveness, is recognised as a key quality measure of care [8]; this is no less the case for children and their families in research.

Unique childhood disease patterns, treatment responses and priorities for intervention acceptability, mean CYP with eye and vision conditions demand research attention [9–12]; evaluating and learning from their research experiences is critical to the quality of this endeavour [8, 13]. This systematic review seeks to understand the paediatric ophthalmic research experiences of CYP, families and research staff.

Planner et al. [14] champion the measurement of research experiences as a necessary feature of quality improvement and a mechanism for ‘patient-centred’ research design. Their scoping review of studies using a standardised measure of experience (1999–2016) found no consensus about how to measure research experience. Work to develop a valid, reliable and acceptable measure for use across trial portfolios is underway [15].

Meanwhile, since 2015, driven by aspirations to involve participants in shaping research delivery through feedback, the National Institute for Health Research (NIHR) via Local Clinical Research Networks (LCRNs) have been measuring research experiences using their ‘Participant in Research Experience Survey’ (PRES). The format of PRES continues to evolve, moving towards national standardisation and away from LCRN variation [16]; up until the 2020/2021 survey, three standardised questions around information provision and general research experience were mandated. In 2019, analysis of national standardised question responses (2018/2019), underpinned development of the ‘Optimising the Participant in Research Experience Checklist’; this identifies five domains significant to shaping positive research experiences, the dimensions of which can be derived from authors’ explanations and examples of each domain [17, 18] (see Table 1). It is striking to note the similarity of these five to domains identified in a study of research retention strategies nearly a quarter of a century ago [19]. To illustrate the similarities, the recommended strategies by Given et al. [19] have been listed alongside the PRES checklist recommendations [18] (see Table 1).

**Table 1:** Five domains which play a significant role shaping participants’ research experiences, identified from participant feedback [17, 18], with similar themed strategies recommended by Given et al. [19] listed for comparison

Domains	Dimensions of domains	Examples provided	Authors’ recommendations	Strategies recommended by Given et al. 1990 [19]
Relationship with research staff	*Extent to which content and character of interactions with research staff meet or exceed participant expectations*	Friendliness Professionalism Knowledge Approachability Helpfulness Respectfulness Responsiveness “Informativeness” “Appreciativeness”	Staff have appropriate training (support) and sufficient time to build relationships	Staff ability to enhance the desire of subjects to participate Staff ability to reflect the knowledge and importance of the project Staff have consideration and concern for others Staff have excellent communication skills Staff have enthusiasm and commitment to the project Pre-trial staff training Continuity of staff to establish trust
Quality and timeliness of information	*Range of formats and media*	Accessibility and breath of formats to suit needs and expectations, e.g. written, verbal, video, online, SMS	Ensure the right information is available in the right place at the right time as participants proceed through the study	Ongoing, open communication (including newsletters, publications and presentations) Communication expectations set from beginning Opportunities for questions and clarifications at every contact point
	*Content*, *accuracy and comprehensiveness*	Pre-consent information (Participant information sheet (PIS)) General information about the health condition ‘Practical’ process information, e.g. appointments, what to expect, where to be, when and how Updates and progress of the study Personal information, e.g. test results Overall study results		
	*Responsiveness*	Responsive to questions		Access and availability of staff Contact number given Prompt response to contact from participants
Engagement with diverse participant motivations	*Extent to which study design/delivery satisfies a range of differing motivations for participation:* ▪Altruistic ▪Health related	*Altruistic* Wanting to help others *Health related* Improved monitoring and care of own condition Hope of improvement in personal medical condition Improved understanding of personal condition	Actively appreciate participants motivations	Project logo and theme to establish project association Project logo and theme used on: letters, gifts (coffee mugs, desk calendar, clock, pens), certificates, questionnaires, newsletters, thank you notes, birthday cards, sympathy cards Statement of appreciation for participants time, contribution and awareness of time and energy they have given to the project
Study organisation	*Extent to which study and intervention design/delivery accommodates participants’ time, monetary and physical/emotional costs*	Appointment scheduling—frequency, length of time, flexibility Waiting times in clinic/between clinics Monetary expenses and how/when incurred/reimbursed Access including location proximity/convenience (any flexibility), travel and parking costs/time/burden Access for those with disabilities (visual impairment, wheelchair access, etc.) Unpleasant side effects or implications of intervention Lifestyle adaptions to accommodate intervention	Involve patients and the public in the earliest stages of the study and intervention design, to identify and mitigate factors which may contribute to adverse participant experiences ‘Walk-throughs’ to carefully consider participants’ pathway from pre-consent, through all study visits	Practice and review simulated study situations Supervision of junior staff by senior staff member Frequent de-briefs for delivery staff to discuss issues and problems and help maintain consistency, enthusiasm and commitment to the project Respect for participants’ time Flexibility in scheduling research visits Follow up if visits missed
Study environment	*Extent to which research environments meet participants’ preferences and expectations*	Noise levels Ambience (calm/busy) Attitudes of others (non-research related) in the environment Availability of refreshments	Carefully consider the effects of the environment participants will be moving through	

## Objectives

The primary objective of this systematic review was to understand what children, young people, their parents and research staff, perceive to support or hinder a positive paediatric eye and vision research participation experience.

The secondary objectives were to:

i) Determine if any adverse or positive *effects* are *perceived* to be related to participation experiences
ii) Identify if any previous *interventions* have been developed or used to *improve* paediatric ophthalmic research experiences

## Methods

This systematic review followed the Centre for Reviews and Dissemination (CRD) guidance for the conduct of healthcare reviews [20] and is reported in accordance with the Preferred Reporting Items for Systematic Reviews and Meta-Analyses (PRISMA) [21]. The protocol was prospectively registered with PROSPERO (Registration Number CRD42018117984, 11 December 2018).

### Search strategy

We searched the electronic databases MEDLINE, Embase, CINAHL, Web of Science, NICE Evidence and The Cochrane Library (CDSR and CENTRAL), from inception until November 2018 and updated the searches in July 2020. Scoping searches identified the most useful search terms and combinations; in order to accommodate our diverse objectives, we designed a sensitive search strategy (see Additional File 1). In addition, key journals were hand-searched, grey literature databases were searched and a focussed search in Google Scholar was conducted (see Additional File 2). References and citations of included studies were screened.

### Study eligibility criteria

Eligibility criteria were defined using the SPIDER (Sample, Phenomenon of Interest, Design, Evaluation, Research type) review tool [22], a tool appropriate for framing qualitative questions [23] (see Table 2). To be included in the review, a study had to explore the paediatric eye and vision research participation experiences/perceptions/views/opinions of CYP and/or their parents and/or research staff. To save *all* ophthalmic studies including CYP being subject to full text screening, we made a pragmatic decision to *only* include studies where outcomes relating to, or discussing research experiences, were reported in the *title* or *abstract*.

**Table 2:** Eligibility criteria

Eligibility criteria
*Inclusion criteria*
* Sample*—any ophthalmic study which included one or more child or young person (birth up to age 16 years); their parents/legal guardians/other carers; ophthalmic research staff, with experience of working in studies which include children and young people up to the age of 16 years
* Phenomenon of interest*—report or discussion of paediatric ophthalmic research participation experience (once enrolled into a study), in the study title or abstract
* Design*—empirical research with *any* study design (including qualitative, quantitative or mixed approaches)
* Evaluation*—views; opinions; perceptions; narratives; scoring or rating
* Research type*—*any* empirical research type
* Publication type*—full text available; published and unpublished research; *any* publication year; English language (resources not available for translation)
*Exclusion criteria*
* Sample*—studies sampling participants in ophthalmic research for adults *only* or *non-*ophthalmic research*;* studies sampling ophthalmology research staff who conduct studies with adults *only* or *non-*ophthalmic studies
* Phenomenon of interest*—studies where phenomenon of interest was *not* experience of research participation
* Research type*—non-empirical research; letters; commentaries; discussion papers; reviews
* Publication type*—studies published in ‘abstract’ form only; studies where full texts were not obtainable (from University of Liverpool, City University of London, University College London or via the Inter Library Loan system which accesses other affiliated universities and the British Library); non-English language

### Study selection

Search results were exported into the Covidence software (© 2020 Covidence) and duplicates removed. Two reviewers (JM, MM) independently screened all title and abstracts to identify potentially relevant articles. Any disagreements were resolved through discussion, with topic expert (JM) taking final decision. The full-text of these articles were then assessed for inclusion independently by two reviewers (JM, ADN) using the eligibility criteria outlined in Table 2. Where lack of eligibility clarity existed, additional information was sought from authors and JC resolved conflicts.

### Quality assessment

The quality assessment tool by Hawker et al. [24] (Additional File 3) was selected to assess the overall quality of the included studies. Due to our eligibility criteria including ‘*any* study design’, Hawker et al. [24] was deemed a suitable tool owing to its ability to cope with quality assessment across a potentially diverse group of empirical studies. Whilst debate continues about the assessment of qualitative methods [23], it was decided that presenting our assessment using these broad criteria would enable transparent decision-making for a range of methodologies. Hawker et al. [24] equated 10 = very poor, up to 40 = good. However, due to a lack of clarity, over how 40 could be reached for a top score (with 9 questions scoring 1–4), MM and JM agreed that for the purposes of this review, the tool would be adapted; 36 would be classed as the maximum score (very poor = 0–9, poor = 10–18, fair = 19–27, good = 28–36). Two reviewers (JM, KCT) appraised included studies and discussed their assessments to reach conclusions on study quality.

### Data extraction and synthesis

Two reviewers (JM, KCT) independently extracted data on study characteristics (author, publication date, country, setting, study sample, setting, experience measure methods) and results from the included studies. The same two reviewers independently conducted a ‘best fit’ synthesis of the included study data against the five domains of the NIHR PRES framework (see Table 1) [25, 26]. This involved discussion of their independent interpretations, to reach judgement on the definitions of the domains, actively seeking disconfirming data (i.e. falling outside the framework), as well as data falling within existing domains, to generate additional domains of research experience, as well as additional dimensions of existing domains. Differences in the synthesis between the two reviewers were resolved through discussion.

## Results

### Searches

**Fig. 1:**
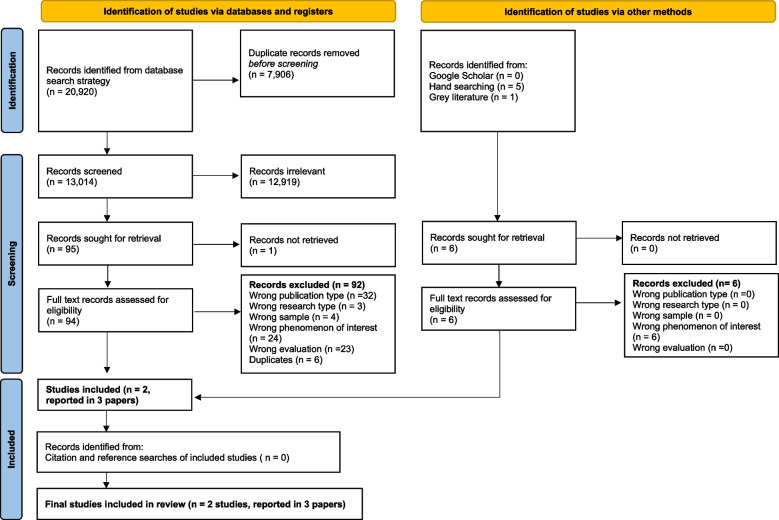
PRISMA flow diagram of screening process

The PRISMA [21] flowchart outlining the screening process is shown in Fig. 1.

Our search strategy identified 20,926 papers, 7906 of which were duplicates. Therefore, 13,020 were screened on title and abstract. This high volume of papers arose from the sensitivity of our search strategy which did not include strings relating to study design or approach. This was to identify studies exploring experiences of research participation, as well as measured effects or interventions to improve experience (see Objectives). Of the 101 papers which progressed to full text review, one was unobtainable. Ninety-eight studies (*n* = 92 identified via databases/registers, *n* = 6 via other methods) were excluded as follows: duplicates (*n* = 6); wrong sample/population (*n* = 4, mostly adult samples); wrong phenomenon of interest (*n* = 30, 7 measuring other experiences, for example of clinical practice, or trial *recruitment* only, or of patient and public involvement activities, 23 measuring experience of the trial *intervention* only). Of these 23 studies which focussed on the experience of the *intervention* only, some evaluated *ophthalmic tests* (*n* = 9) such as vision screening, intraocular pressure (IOP) measurement, perimetry, fundoscopy assessments and ptosis assessments, and others (*n* = 14) evaluated *treatments* for ophthalmic conditions such as amblyopia, allergic conjunctivitis and myopia. How children and young people experience interventions is important (including what measure is used and how the data is reported). However, after scrutiny by the review team, it was agreed that the broad experiences children had participating in these studies was not measured (therefore, they were excluded); wrong evaluation (*n* = 23, did not measure experiences); wrong research type (*n* = 3, not empirical); wrong publication type (*n* = 32, no published datasets—protocols, studies in progress or abstract only). Two studies (reported in three papers) met the inclusion criteria: Dias et al. [27], Buck et al. [28] and Clarke et al. [29] (with Buck et al. [28] reporting results pertaining to this review more comprehensively than Clarke et al. [29]).

### Included study characteristics

Key characteristics and research participation experience measure methods for the two included studies are presented in Additional File 4.

### Quality assessment

Quality assessments ratings of the two included studies are presented in Additional File 3. Both studies were graded ‘good’ (‘good’ range 28–36), with scores of 30 [28] and 34 [27]. Despite both studies being ‘good’, we were conscious of the following limitations when synthesising their findings. For Buck et al. [28], the wide range of data collection time points defocused the purpose of their evaluation by not accounting for altered perceptions over time. It would be interesting to know how insights changed from those interviewed just after enrolment, compared to those 10 weeks later, for example. In addition, their interviews were not recorded ad verbatim with transcription; instead, notes were inputted into a computer whilst conducting the interview. This may be what led to minimal exemplar quotes and a lack of detail in the results reported. More information on prominence of the different themes identified would have enhanced the results section, together with details to explain certain aspects (for example the meaning of ‘communication’ in their results Table 3). The sample relevant to this review was small (*n* = 14); little rationale is given about the sample size or the demographic details of the interviewees.

For Dias et al. [27], the questionnaire itself was an adapted version of a survey used in the Framingham Heart Study [30]; no information is given about how either questionnaire was designed. Whether the items evaluated in the questionnaires *were* aspects of care, which families would consider important to their experiences, remains unknown, since there is no mention of involving families (or staff) in either. In addition, using a closed-questionnaire format only did not allow for ‘any other comments’ or for families to highlight ‘any other’ aspects of their research experiences not listed. Dias et al. [27] also used their surveys for parents to rate the aspects of the study ‘important to retention’. However, the results presented do not share the ratings, instead focussing on the comparison between family and staff opinions.

### Primary outcome

No data were identified where CYP were directly consulted on their own experiences of research participation. The perspectives of 425 parents of children between the ages of 6 months and 16 years were sought by Dias et al. [27] and Buck et al. [28] combined. Dias et al. [27] complemented parents’ perspectives by collecting the views of 35 research staff, however seeking staffs’ perceptions of families’ trial experiences, rather than their own perspectives or experiences.

Dias et al. [27] used a survey design and focussed in general on how families liked a lot more aspects of the COMET study [31] than staff thought they would. Buck et al. [28] used telephone interviews to explore which aspects of the SamExo study [32] parents deemed acceptable.

The results for our primary objective are presented below, under the domains of the PRES framework detailed in Table 1. Table 3 indicates which studies contributed to each domain.

**Table 3:** Summary of domains contributions (‘X’ represents study contribution)

Study ID	**Domains**				
	**Relationships with research staff**	**Quality and timeliness of information**	**Engagement with diverse participant motivations**	**Study organisation**	**Study environment**
Dias et al. [27]	X	X	X	X	
Buck et al. [28]		X	X	X	

#### Relationship with research staff

Dias et al. [27] measured the extent to which parents ‘*liked*’ the following characteristics of staff: staff response to questions, friendliness, quality of eye care, positive encouragement, seeing the same staff at each visit. In alignment with the PRES framework (Table 1), this domain stood out as supporting positive experiences and being *‘liked*’ by *all* 411 parents; 95–98% awarding the highest rating (*‘liked a lot*’) to *staff friendliness*,* response to questions*,* quality of eye care *and* positive encouragement* [27]. It is worth noting that as quality assurance measure embedded within the COMET study, staff received training on the importance of ‘prompt responses to questions’ and had clear protocols for problem solving. Staff *under*estimated the extent to which parents valued each ‘staff characteristics’.

In Buck et al. [28], parents suggested ‘communication’ was needed to make the trial *more* acceptable. However, from their reporting, it is difficult to identify what this relates to potentially ‘staff characteristics’ or ‘information’ or ‘study organisation’.

#### Quality and timeliness of information

When parents talked about what would make the SamExo study more acceptable [28], the majority of suggestions related to the ‘study information’. In alignment with the PRES framework (Table 1), parents’ preferences focused around the format, content and comprehensiveness of information. One parent suggested simplification of the Patient Information Sheet (PIS), with another who felt the randomisation explanation was ‘too detailed’. In relation to content of the information, despite most parents being ‘satisfied’, Buck et al. [28] reported parental concerns about the following missing or inadequate content: the operation (intervention) itself (e.g. how long it would take), success rates and outcomes, general information about the condition itself and the need for randomisation in the study design. Validating this deficiency, some parents revealed a lack of knowledge or understanding for the randomisation during their interviews. A shortfall of information about ‘risks’ was also identified, with one parent commenting about the importance of the ‘NHS’ providing this information rather than having to rely on ‘online’ information which could be “dangerous”. Finally, parents requested extra information about the ‘costs of participation’, which the study authors assume to be solely related to ‘monetary’ costs. It is worth remembering the range of time points the interviews were conducted in Buck et al. [28] and considering how parents might view the information differently at 10 weeks compared to 2 days into trial.

The focus of information provision by Dias et al. [27] related to ‘updates and progress’, where they evaluated parents’ views of their newsletters (93% of parents ‘*liked’*) and appointment reminders (telephone calls and postcards prior to visits) (98% of parents ‘*liked’*). Details of the newsletters content, format or frequency are not reported. Staff *under*estimated the extent to which these updates were ‘*liked’*.

#### Engagement with participants’ diverse motivations for participation

##### Altruistic

Buck et al. [28] aligned with the PRES framework (Table 1) by identifying some parents altruistic motivations for ‘doing their bit’ for research, though this was never their sole motivation. Dias et al. [27] categorised their newsletters as ‘reinforcements’; potentially aiming to ‘reinforce’ or nurture participating families’ altruistic motivations; though we are not privy to the newsletter content.

##### Health related

Improved monitoring and care of own condition: similarly to PRES (Table 1), both Buck et al. [28] and Dias et al. [27] report data suggesting that parents were motivated by an expectation that the best or ‘expert’ care, superior to regular clinical care, could be achieved through participation in research. Parents in Buck et al. [28] report being reassured that their child would be monitored. In Dias et al. [27], very high percentages of parents ‘*liked*’ the ‘quality of eye care’ (99%) and ‘completeness of eye exam’ (99%). Ninety-seven percent of parents ‘*liked*’ the ‘association with the College of Optometry’ and ninety-nine percent of parents ‘*liked*’ being ‘part of a nationwide study’, both of which could potentially signal to parents a nationwide availability of high quality and trustworthy care (with a standard of care set by the College of Optometry).

Hope of improvement in personal medical condition: for this dimension, in direct contrast to participants being motivated to participate through the hope of improvement, one parent [28] raised how parents may be ‘put off’ because the study was about ‘the eyesight’. No rationale is given, though the statement implies this parent felt ‘the eyesight’ was either an especially important aspect of their child’s health or an especially vulnerable aspect of their physiology. Either way, the sense is given that the threat of a potential *decline* in personal medical condition, through research participation, may act as a *de-*motivating factor.

##### Opportunity for (relatively) flexible treatment options

This *new* sub-dimension identified in Buck et al. [28] was *added* to the PRES framework (Table 4), based on parents’ lack of preference or timeframe for treatment, which thereby increased their willingness or motivation to join a *randomised* design trial. Parents said they had ‘nothing to lose’—they were happy to have surgery (the study intervention) but also happy to wait (until *or* if a constant strabismus appeared to be developing, *or* if parents request surgery and the responsible clinical team agreed that this was appropriate, *or* at the end of the trial if randomised to ‘active monitoring’ arm [32]). Some parents mentioned that they would not be ‘denied’ surgery, so it was a question of ‘when’ they would have surgery not ‘if’ they would have surgery. Therefore, if joining the study resulted in a delay of surgery, this was an acceptable outcome to some parents whose child had participated. In addition, parents felt reassured they could change their mind about participation (withdrawing from study). Parents were therefore motivated for their child to participate by the variety, flexibility and potentially reversible (withdrawing from study) treatment options which study participation provided.

##### Opportunity to relinquish personal responsibility for unforeseen effects whilst trying new treatment

This second *new* sub-dimension *added* to the framework (Table 4) was derived from one parent who described wanting ‘someone else’ to make the decision for them [28]. Joining a randomised controlled trial (RCT) was described as a ‘positive’, through being exonerated of personal responsibility for a treatment decision by surrendering to random allocation. In the COMET trial [27], parents had to agree to *accept* the random assignment of treatment and continue for at least 3 years: their ‘COMET commitment’. Eighty-nine percent of parents ‘*liked a lot*’, the ‘COMET commitment’, though we do not learn any details about why. Potentially, like the parent in Buck et al. [28], parents in the COMET trial also liked the opportunity to *surrender control* of treatment. It would have been interesting to gain a deeper understanding of why parents ‘*liked*’ this commitment and indeed explore the feelings of the CYP themselves.

##### Material incentives designed in by researchers

This third *additional* sub-dimension (Table 4) was generated from data collected by Dias et al. [27] and related to material incentives, designed into studies by researchers. For Dias et al. [27], ‘thank you’ materials were classed by the authors as ‘incentives’ (movie passes, gift certificates to local attractions, T-shirts, photo frames and other items with the COMET logo, other small age-specific toys and trinkets) plus free glasses (including sports glasses) and free repair/maintenance of glasses; these were all incorporated into the study design and *liked* by all but 2% of parents.

#### Study organisation

Included study data aligned well with PRES (Table 1) in terms of organisational aspects appreciated or disparaged by participants. Within Dias et al. [27], families appreciated the flexibility and convenience of appointment times, which included evenings and weekends. When asked whether they liked the length of study (3 years), the majority did, though a small minority did not. Equally, a small minority gave less positive rankings about the ‘location and access to the study site’ [27]; there were four sites, though no details are given of which site(s) posed the problem or why. Staff *over*estimated the number of parents who *dis*liked the study length and centre access.

In relation to accommodating anticipated ‘costs’ encountered through participation, as highlighted earlier, there was a mention of *un*anticipated *costs* in Buck et al. [28]; no details are given as to whether the costs incurred were felt reasonable. Within Dias et al. [27], related to the physical and emotional costs of participation, families and staff rated the eye drops (Anaesthetic, Tropicamide, Fluorescein) required for eye examinations as the *least* popular feature of the study. Excluding the physical and emotional costs of study ‘interventions’, the PRES framework (Table 1) only lightly touches on the physical and emotional costs of ‘research participation’, in quotes which relate to the emotional burden of ‘waiting’, for example. Perceived burden of eye drops [27] is an important consideration for eye and vision research, where the use of drops which sting for assessments is common. For CYP in particular, it is ethically important to pay special attention to the emotional and physical burden of trial participation. Therefore, *adding* this example of ‘*assessment* burden/discomfort/distress’ to this dimension builds in necessary depth to the ‘study organisation’ domain (Table 4).

**Table 4:** Five key PRES domains [17, 18] with additions (bold) from synthesis of included studies results

Domains	Dimensions of domains	Examples provided	Authors’ recommendations
Relationship with research staff	*Extent to which content and character of interactions with research staff meet or exceed participant expectations*	Friendliness Professionalism Knowledge Approachability Helpfulness Respectfulness Responsiveness “Informativeness” “Appreciativeness”	Staff have appropriate training (support) and sufficient time to build relationships
Quality and timeliness of information	*Range of formats and media*	Accessibility and breath of formats to suit needs and expectations, e.g. written, verbal, video, online, SMS	Ensure the right information is available in the right place at the right time as participants proceed through the study
	*Content*, *accuracy and comprehensiveness*	Pre-consent information (Participant information sheet (PIS)) General information about the health condition ‘Practical’ process information, e.g. appointments, what to expect, where to be, when and how Updates and progress of the study Personal information, e.g. test results Overall study results	
	Responsiveness	Responsive to questions	
Engagement with diverse participant motivations	*Extent to which study design/delivery satisfies a range of differing motivations for participation:* ▪Altruistic ▪Health related ▪**Opportunity for (relatively) flexible treatment options** ▪**Opportunity to relinquish personal responsibility for unforeseen effects whilst trying new treatment** ▪**Material incentives designed in by researchers**	*Altruistic* Wanting to help others *Health related* Improved monitoring and care of own condition Hope of improvement in personal medical condition Improved understanding of personal condition ***Opportunity for (relatively) flexible treatment options*** **Join randomised controlled trial (RCT) with a chance of receiving trial intervention, always with the freedom to withdraw (and potentially seek treatment off study)** **Join RCT and be randomised to non-intervention, but still have the opportunity for intervention later** ***Opportunity to relinquish personal responsibility for unforeseen effects whilst trying new treatment*** **RCT – willingly surrender to random allocation of treatment** ***Material incentives designed in by researchers*** **Thank you materials** **Condition specific equipment/servicing of equipment**	Actively appreciate participants motivations
Study organisation	*Extent to which study and intervention design/delivery accommodates participants’ time, monetary and ****physical/emotional**** costs*	Appointment scheduling—frequency, length of time, flexibility Waiting times in clinic/between clinics ***Assessment* burden/discomfort/distress** Monetary expenses and how/when incurred/reimbursed Access including location proximity/convenience (any flexibility), travel and parking costs/time/burden Access for those with disabilities (visual impairment, wheelchair access, etc.) Unpleasant side effects or implications of intervention Lifestyle adaptions to accommodate intervention	Involve patients and the public in the earliest stages of the study and intervention design, to identify and mitigate factors which may contribute to adverse participant experiences ‘Walk-throughs’ to carefully consider participants’ pathway from pre-consent, through all study visits
Study environment	*Extent to which research environments meet participants’ preferences and expectations*	Noise levels Ambience (calm/busy) Attitudes of others (non-research related) in the environment Availability of refreshments	Carefully consider the effects of the environment participants will be moving through

#### Study environment

No data was identified related to this domain of the framework.

### Secondary outcomes

No data were identified to meet our secondary outcomes. The exploration by Dias et al. [27] of aspects ‘important to retention’ did not measure proven effects or associations between families’ preferences and retention.

## Discussion

We found no evidence capturing CYP’s own experiences of ophthalmic research participation (objective 1) nor measured *effects* of participation experiences or *interventions* designed to *improve* paediatric ophthalmic research experiences (objectives 2). Two studies were identified which captured parents’ experiences of their child’s ophthalmic research participation (objective 1).

From a parent perspective therefore, our primary review outcome both concurs with and expands on previous research participation experience evaluations [17, 18]. The extent to which the dimensions and domains of research care (Table 4) are accommodated in research design and delivery may enhance (or undermine) participants’ and their families’ experiences accordingly.

Like other adults [17, 18], parents’ experiences confirmed the important role of positive staff interactions [27], comprehensive and accessible information provision [27, 28] and engagement with participants’ personal motivations for taking part; such as hope for improved monitoring and care [27, 28] or material benefits one can receive as part of a study design (e.g. free maintenance and provision of glasses [27]). Parents were also motivated by flexible and potentially reversible (withdrawing from study) treatment options available via trial participation [28], including the option, afforded by intervention randomisation, to relinquish personal responsibility for a treatment decision [27, 28]. Our synthesis added to the understanding of concerns about health deterioration as a barrier, specifically that this may be especially salient in the context of ophthalmic research where ‘eyesight’ is regarded as a particularly precious and fragile resource [28]. Aligning with the PRES framework (Table 1), the degree to which study logistics were well organised, flexible, convenient and accessible, was important [27]. In particular, our data flagged a need for greater attention to the physical and emotional burden of participation, for example, discomfort associated with research assessments [27]; this perhaps is a function of the paediatric population and the associated ethical considerations [33].

Re-addressing the validity of the survey data, we remember authors provided no account of how instruments were developed nor mention of exploratory evidence about participants’ concerns and priorities to underpin robust survey design. That said, the list of ‘staff characteristics’ posed [27] was broadly similar to examples given in PRES (Table 1). Additions were ‘positive encouragement’ and ‘seeing the same staff at each visit’, which were also staff characteristics highlighted by Given et al. [19] and which we know young people value in the context of routine long-term care [34]. ‘Positive encouragement’ may be particularly pertinent for CYP participating in eye and vision research, where tests and assessments can demand sustained focus and stillness and are often scheduled one after the other in trial protocols, although equally alongside ‘encouragement’, focusing protocol design around the needs of CYP and thereby avoiding extended sequences of testing should also be a consideration.

As discussed in the assessment of quality, a more thorough qualitative design [28], or the inclusion of open text data collection [27], may have provided more understanding about *why* aspects of research participation were acceptable or ‘*liked*’. In some of the excluded studies (excluded on the basis of *only* evaluating ‘experience of the ophthalmic intervention’), the richness in data collected through qualitative methods led to greater understanding of experiences with some important clinical implications. For example, Carrara et al. [35] who conducted interviews to capture parental perspectives on their newborn visual function test collected such rich data, a change of future practice followed, despite only being raised by 1% of their population (not handling the baby with one arm during the test). In other studies where *formal* collection of *verbatim* comments were collected, the understanding of results was also significantly enhanced, for example, Patel et al. [36] where in addition to a difficult rating scale for static perimetry, comments by children explained that it was the *rapid* rate and *intensity* of stimuli presentation, which raised the difficulty rating, despite being shorter in duration. Without formal collection or systematic analysis, ad hoc comments are less reliable (despite sometimes being added to concluding statements, e.g. Martin ([37] p676) “the children found it rather entertaining”).

Parents’ views, in the context of their important role supporting treatment or research participation, are crucial; but they do not necessarily dovetail with the priorities and concerns of CYP themselves [34, 38–41]. Though omitted in the included studies, it was heartening to see in a small number of the excluded studies (for example [36, 42], excluded due to *only* evaluating the ‘experience of the intervention’), attention given directly to CYP’s own perceptions. The United Nations Convention on the Rights of the Child (UNCRC) [43] asserts children’s rights to both “the highest attainable standard of health and to facilities for treatment” and to being asked their perspectives on matters which affect them; a robust evidence base for paediatric ophthalmic healthcare requires attention to the research experiences of CYP themselves and validated measures to monitor this.

Dias et al. [27] spent much time comparing families’ experience ratings with estimates made by staff. Whilst the benefit of staff ‘estimates’ was not immediately obvious, it was interesting to note a broad finding that staff consistently *under*estimated aspects of the experience families ‘*liked’* (for example, staff characteristics, newsletters, appointment reminders). As Dias et al. [27] highlighted, it is important staff have accurate knowledge of research care aspects families value, in order to be able to correctly channel resources and focus (for example, writing or disseminating newsletters) to achieve positive participation experiences. The other notable discrepancy was how staff consistently *over*estimated *negative* ratings by families (for example, study centre access, selections of frames and eye drops). With ‘eye drops’, for example, there was a significant difference with 22% of families ‘*disliking*’ the eye drops compared with the staff estimate that 83% families ‘*disliked*’ eye drops; in addition, 14% families unusually did not answer the eye drops question. The explanation for this tension is hindered by data collection methods limited to closed questionnaire surveys. This points to the value of in-depth qualitative approaches to gain insights into experiences, where context is collected to help explain and interpret findings. Despite efforts to gain honest reporting (responses being sent *direct* to the coordinating centre and anonymity for staff), these discrepancies of opinion could potentially highlight that families still felt uncomfortable reporting negative experiences, or may simply represent differing perspectives, emphasising the importance and value of collecting perspectives from various stakeholders, in particular CYP themselves.

The added value from collecting multiple perspectives was also evident in some of the excluded studies (excluded for *only* evaluated the ‘experience of the intervention’), for example, Carrara et al. [35], where parents raised concerns regarding testing newborns so ‘early’; in contrast, staff emphasised the *need* for an early test, to increase the chances of the baby being awake and the test therefore being *easier* to conduct. Similarly, Patel et al. [36] triangulated the perspectives of CYP with their parents and the examiner. Their methods, using an Examiner Based Assessment of Reliability (EBAR) score, and comparing scores with the children’s difficulty rating, led to an interesting findings that *no* relationship was detected between the two. They found it was not always the tests children perceived as ‘hard/difficult’, which were unreliable nor the ‘easy’ tests which were reliable.

### Strengths and limitations

Whilst the sensitivity and scope of our literature search means we are confident no interventions to ‘*improve* the paediatric experience of eye and vision research participation’ have been developed and tested to date, it became apparent that excluding studies on the basis of ‘no mention of an ‘experience’ outcome within the title or abstract’ could be a potential limitation. Sometimes, where the ‘experience’ evaluation was *not* the *main* focus of a study, less formal reporting occurred; this also highlights a wider issue around the reporting of ‘experience’ measures and the varied levels of the importance placed upon this type of outcome within studies.

In addition, ‘experience’ is not well defined in the literature; a wide range of terminology and measures were identified to infer ‘experience’ outcomes (see Additional File 5). A similar variety of terms and measures was identified by Sekhon et al. [44] when reviewing the concept and definition of ‘acceptability’ in relation to health care interventions. They found a mixture of *self-report* measures (satisfaction measures, experiences or perceptions, interviews, side effects) and *observed behaviour* measures (dropout rates, reason for discontinuation, withdrawal rates).

### Implications for future research

The evidence to understand paediatric eye and vision research experience is scarce; most importantly, it fails to include the voices of CYP themselves. Future research to expand the evidence base, using methodology accessible and acceptable to involve CYP, is recommended to:

- Better understand paediatric eye and vision research participation experiences;
- Direct how teams can maximise what enables CYP to have positive experiences and minimise what leads to CYP having poor experiences, in the design and delivery of eye and vision research;
- Explore any *effects* of positive and negative experiences, including potential relationships with recruitment and retention to studies.

Equally, no validated instruments were used to measure experiences. Our review builds on the recommendations of Planner et al. [14] with the following suggestions:

- That multiple perspectives are collected, including the voice of CYP themselves, in a format that is age appropriate and meaningful;
- The inclusion of all stakeholders in the design of experience measures, to ensure instruments address aspects of research experiences important to participants;
- That a qualitative component is included to ensure the richness of data required to enable full understanding;
- That the dominance of the ‘experience of the *intervention*’, in the context of paediatric ophthalmic research participation experience, is further explored. Currently, the literature is mainly limited to this type of experience evaluation (which was excluded from this review); it is important to decipher which experience domains are the most important and impactful to stakeholders;
- That further review is conducted of paediatric ophthalmic ‘*intervention*’ experience measures. Twenty-three papers were excluded from this review for measuring ‘experience of the trial *intervention only*’; though excluded, a similar paucity of robust, validated, child-friendly experience measures was noted.
- That consideration is given to the definition and indexing of ‘experience’ terminology, together with expectations for formal reporting for ‘experience’ outcomes. This would help researchers consider the purpose of their experience evaluation(s) and the type of measure used.

## Conclusion

Understanding the experiences of CYP taking part in eye and vision research is important to trial integrity; findings can direct improvements, enhancing research design and delivery and promoting quality, credible, child-centred research. However, the current limited evidence base only captures the experiences of parents and a small number of staff; the voices of CYP and their evaluations of their own experiences are missing. Our review adds detail to the current evidence base on aspects of research care pivotal to experiences; whether these additions may or may not be limited to eye and vision research is unknown. Further investigation, involving CYP, could expose a unique perspective, which could both inform the way research ‘experience’ is measured, and lead to improvements in the quality of paediatric ophthalmic research care; which in turn will maximise the visual outcomes for CYP in the future.

## Supplementary Information

**Additional file 1.** Search Strategy.

**Additional file 2.** Key journals hand-searched.

**Additional file 3.** Quality scoring using an adapted Hawker et al. (2002) assessment tool.

**Additional file 4.** Summary of papers which evaluate paediatric eye and vision research experiences.

**Additional file 5.** Terminology and measures identified to infer ‘experience’ outcomes.

## Data Availability

All datasets used/or analysed during the current review are available from the corresponding author on reasonable request.

## Abbreviation

CYP: Children and Young People
