# Supplementary material for: Paediatric eye and vision research participation experiences: a systematic review
Source: Trials. 2023 Jan 28;24:66. doi: 10.1186/s13063-022-07021-1 (PMC9883950; doi:10.1186/s13063-022-07021-1)
Supplement: Supplementary file 1 — Additional file 1. Search Strategy. [file 13063_2022_7021_MOESM1_ESM.docx]

**Additional File 1**

**Search Strategy**

Database: Ovid MEDLINE(R)

Search Strategy:

--------------------------------------------------------------------------------

1 exp OPHTHALMOLOGY/ (21601)

2 exp EYE/ (331542)

3 exp Ophthalmologic Surgical Procedures/ (103718)

4 ophthalm*.ti,ab. (93261)

5 eye*.ti,ab. (344664)

6 sight.ti,ab. (11159)

7 vision.ti,ab. (101036)

8 glaucoma.ti,ab. (52262)

9 conjuncti*.ti,ab. (121657)

10 uveitis.ti,ab. (16469)

11 macula*.ti,ab. (53979)

12 oedema.ti,ab. (24902)

13 edema.ti,ab. (100303)

14 strabismus.ti,ab. (10547)

15 squint.ti,ab. (1557)

16 astigmati*.ti,ab. (10126)

17 myopi*.ti,ab. (18917)

18 hypermetropia.ti,ab. (611)

19 trachoma.ti,ab. (3823)

20 ocular.ti,ab. (114541)

21 cornea*.ti,ab. (86168)

22 retin*.ti,ab. (272326)

23 scler*.ti,ab. (192951)

24 vitre*.ti,ab. (38735)

25 iris.ti,ab. (18431)

26 pupil.ti,ab. (12455)

27 orbit*.ti,ab. (93738)

28 chorod*.ti,ab. (23)

29 intraocular.ti,ab. (60638)

30 intra-ocular.ti,ab. (1822)

31 extraocular.ti,ab. (6827)

32 extra-ocular.ti,ab. (569)

33 monocular.ti,ab. (8582)

34 oculo*.ti,ab. (20036)

35 oculi.ti,ab. (3021)

36 optic*.ti,ab. (338948)

37 visual*.ti,ab. (550983)

38 or/1-37 (1985739)

39 exp CHILD/ (1795171)

40 exp INFANT/ (1078665)

41 exp ADOLESCENT/ (1894393)

42 pediatric*.ti,ab. (254409)

43 paediatric*.ti,ab. (56067)

44 child*.ti,ab. (1265321)

45 infant*.ti,ab. (373940)

46 adolescen*.ti,ab. (245230)

47 youth*.ti,ab. (63608)

48 teen*.ti,ab. (27852)

49 or/39-48 (3795665)

50 38 and 49 (279910)

51 exp "PATIENT PARTICIPATION"/ (23092)

52 exp "RESEARCH SUBJECTS"/ (18057)

53 exp "PATIENT SELECTION"/ (60077)

54 ((participat* or enrol* or involve* or recruit* or retention or "taking part" or "took

part" or experience*) and (research or study or studies or trial*)).ti. (34866)

55 ((participat* or enrol* or involve* or recruit* or retention or "taking part" or "took

part" or experience*) adj3 (research or study or studies or trial*)).ab. (230915)

56 ((participat* or enrol* or involve* or recruit* or retention or "taking part" or "took

part") and (experience* or perception* or view* or opinion* or assess* or choice*or

barrier* or facilitator* or satisf* or acceptab*)).ti. (5923)

57 ((participat* or enrol* or involve* or recruit* or retention or "taking part" or "took

part") adj3 (experience* or perception* or view* or opinion* or assess* or choice*or

barrier* or facilitator* or satisf* or acceptab*)).ab. (26973)

58 51 or 52 or 53 or 54 or 55 or 56 or 57 (374507)

59 50 and 58 (6719)

60 limit 59 to english language (6240)
