# Supplementary material for: Paediatric eye and vision research participation experiences: a systematic review
Source: Trials. 2023 Jan 28;24:66. doi: 10.1186/s13063-022-07021-1 (PMC9883950; doi:10.1186/s13063-022-07021-1)
Supplement: Supplementary file 2 — Additional file 2. Key journals hand-searched. [file 13063_2022_7021_MOESM2_ESM.docx]

**Additional File 2:**

**Key journals hand-searched**

- **Ophthalmology** (Wang et al., 2019) identified
- **Clinical Trials**
- **Trials** (Herbison et al., 2016, Gao et al., 2018) identified
- **JAMA Ophthalmology**
- **Journal of Pediatric Ophthalmology and Strabismus** (Smith et al., 2019) identified
- **PLOS One**
- **Optometry and Vision Science** (Huang et al., 2019) identified
- **British Journal of ophthalmology**
- **Pilot and feasibility studies**
- **ACTA Ophthalmologica**
- **Patient Experience Journal**
- **Journal of Patient Experience**

**Grey literature search**

- **BASE Bielefeld Academic Search Engine**
- **EThOS** (Aslam, 2012) identified
- **Open Grey**
- **City Research Online**

**Google scholar searches**

**a: Run in August 2019**

**b: Run in Nov 2020 - ‘Since 2019’**

|  | **Terms** | **Number identified** | **Number included** |
| --- | --- | --- | --- |
| 1a | allintitle:  research eye children | 41 | 0 |
| **1b** | **allintitle:**  **research eye children** | **1** | **0** |
| 2a | allintitle:  research eye pa**e**diatric | 1 | 0 |
| **2b** | **allintitle:**  **research eye paediatric** | **0** | **0** |
| 3a | allintitle:  research eye pediatric | 8 | 0 |
| **3b** | **allintitle:**  **research eye pediatric** | **0** | **0** |
| 4a | allintitle:  research ophthalmology children | 8 | 0 |
| **4b** | **allintitle:**  **research ophthalmology children** | **0** | **0** |
| 5a | allintitle:  research ophthalmology pa**e**diatric | 0 | 0 |
| **5b** | **allintitle:**  **research ophthalmology paediatric** | **0** | **0** |
| 6a | allintitle:  research ophthalmology pediatric | 10 | 0 |
| **6b** | **allintitle:**  **research ophthalmology pediatric** | **1** | **0** |

ASLAM, T. M. 2012. *An exploration of novel applications and investigations at the interface of computing and ophthalmology.* Heriot-Watt University.

GAO, T. Y., GUO, C. X., BABU, R. J., BLACK, J. M., BOBIER, W. R., CHAKRABORTY, A., DAI, S., HESS, R. F., JENKINS, M. & JIANG, Y. 2018. Effectiveness of a binocular video game vs placebo video game for improving visual functions in older children, teenagers, and adults with amblyopia: a randomized clinical trial. *JAMA ophthalmology,* 136**,** 172-181.

HERBISON, N., MACKEITH, D., VIVIAN, A., PURDY, J., FAKIS, A., ASH, I. M., COBB, S. V., EASTGATE, R. M., HAWORTH, S. M. & GREGSON, R. M. 2016. Randomised controlled trial of video clips and interactive games to improve vision in children with amblyopia using the I-BiT system. *British Journal of Ophthalmology,* 100**,** 1511-1516.

HUANG, J., MUTTI, D. O., JONES-JORDAN, L. A. & WALLINE, J. J. 2019. Bifocal & Atropine in Myopia Study: Baseline Data and Methods. *Optometry and Vision Science,* 96**,** 335-344.

SMITH, K. A., ARNOLD, A. W., SPRANO, J. H., ARNOLD, S. L. & ARNOLD, R. W. 2019. Performance of a Quick Screening Version of the Nintendo 3DS PDI Check Game in Patients With Ocular Suppression. *Journal of pediatric ophthalmology and strabismus,* 56**,** 234-237.

WANG, C. Y., ZHANG, G., TANG, B., JIN, L., HUANG, W., WANG, X., CHEN, T., ZHU, W., XIAO, B. & WANG, J. 2019. A Randomized Noninferiority Trial of Wearing Adjustable Glasses versus Standard and Ready-made Spectacles among Chinese Schoolchildren: Wearability and Evaluation of Adjustable Refraction III. *Ophthalmology*.
