# Supplementary material for: Paediatric eye and vision research participation experiences: a systematic review
Source: Trials. 2023 Jan 28;24:66. doi: 10.1186/s13063-022-07021-1 (PMC9883950; doi:10.1186/s13063-022-07021-1)
Supplement: Supplementary file 3 — Additional file 3. Quality scoring using an adapted Hawker et al. (2002) assessment tool. [file 13063_2022_7021_MOESM3_ESM.pdf]

Additional File 3:

Quality scoring using an adapted Hawker et al. (2002) assessment tool

Study ID	Dias et al. (2005) [1]	Buck et al. (2015) [2]
**Hawker et al. (2002) scoring system**	Good = 4<br>Fair = 3<br>Poor = 2<br>Very Poor = 1	Good = 4<br>Fair = 3<br>Poor = 2<br>Very Poor = 1
**1. Abstract and title: Did they provide a clear description of the study?**	4	4
**2. Introduction and aims: Was there a good background and clear statement of the aims of the research?**	4	4
**3. Method and data: Is the method appropriate and clearly explained?**	3	2
**4. Sampling: Was the sampling strategy appropriate to address the aims?**	4	2
**5. Data analysis: Was the description of the data analysis sufficiently rigorous?**	4	4
**6. Ethics and bias: Have ethical issues been addressed, and what has necessary ethical approval gained? Has the relationship between researcher and participants been adequately considered?**	4	4
**7. Results: Is there a clear statement of the findings?**	3	3
**8. Transferability or generalizability: Are the findings of this study transferable (generalizable) to a wider population?**	4	3
**9. Implications and usefulness: How important are these findings to policy and practice?**	4	4
**Adapted Hawker et al. (2002) grading:**<br>**Very Poor = 0 - 9 Poor = 10 - 18 Fair = 19 - 27 Good = 28 - 36**	**34 = Good**	**30 = Good**

BUCK, D., HOGAN, V., POWELL, C. J., SLOPER, J. J., SPEED, C., TAYLOR, R. H., TIFFIN, P. & CLARKE, M. P. 2015. Surrendering control, or nothing to lose: parents' preferences about participation in a randomised trial of childhood strabismus surgery. *Clinical Trials,* 12**,** 384-393.

DIAS, L., SCHOENFELD, E., THOMAS, J., BALDWIN, C., MCLEOD, J., SMITH, J., OWENS, R. & HYMAN, L. 2005. Reasons for high retention in pediatric clinical trials: comparison of participant and staff responses in the Correction of Myopia Evaluation Trial. *Clinical Trials,* 2**,** 443-452.

HAWKER, S., PAYNE, S., KERR, C., HARDEY, M. & POWELL, J. 2002. Appraising the Evidence: Reviewing Disparate Data Systematically. *Qualitative Health Research,* 12**,** 1284-1299.
