# Supplementary material for: Paediatric eye and vision research participation experiences: a systematic review
Source: Trials. 2023 Jan 28;24:66. doi: 10.1186/s13063-022-07021-1 (PMC9883950; doi:10.1186/s13063-022-07021-1)
Supplement: Supplementary file 4 — Additional file 4. Summary of papers which evaluate paediatric eye and vision research experiences. [file 13063_2022_7021_MOESM4_ESM.pdf]

Additional File 4: Summary of papers which evaluate paediatric eye and vision research experiences

Author, date, country	Ocular morbidity	Aim	Setting	Perspective	Sample	Data collection	Experience measure	Data analysis
Dias et al., 2005, USA	Myopia	1.To evaluate the degree to which aspects of study participation were 'liked/disliked'<br><br>2. To compare perspectives on the importance to aspects of study participation to families' continued participation (retention)	4 Centres:<br>1) University of Alabama School of Optometry in Birmingham, Alabama;<br>2) New England College of Optometry in Boston, Massachusetts;<br>3) University of Houston College of Optometry in Houston, Texas;<br>4) Pennsylvania College of Optometry in Philadelphia, Pennsylvania<br><br>Coordinating Centre located at Stony Brook University, New York	▪ Parents<br>▪ Staff (clinical centre staff including optometrists, clinic coordinators and opticians)	▪ 411 (88%) parents whose child (aged 6-11 years) had participated in the COMET Study (Gwiazda et al., 2003)<br><br>Ethnicity of respondent's participating **child**: African American 25.6%, Asian 7.8%, Hispanic 14.1%, Mixed 5.4% and White 47.2%<br><br>Study Centre respondent's participating **child** attended: Alabama 28%; Boston 24.1%; Houston 26%; Philadelphia 21.9%<br><br>▪ 35 (74%) staff (current and former; working on COMET study)<br><br>No further details given for staff	Non-validated, study specific 19-item questionnaire<br><br>Distributed at 3.5 year follow visit, completed on site and returned in sealed envelope OR completed at home and sent directly to coordinating centre in stamped addressed envelopes (SAEs). Families who did not return the initial survey were mailed a second survey with a SAE.<br><br>Blank surveys mailed to staff; mailed directly back to coordinating centre in SAE.<br>Staff surveys were anonymous so responses could not be tracked.	Participants indicated level of preference (1- disliked a lot to 4 - liked a lot) across 4 domains:<br>▪ Staff characteristics (response to questions, friendliness, quality of eye care, positive encouragement, seeing the same staff at each visit)<br>▪ Operational aspects (appointment reminders, convenience of appointments, location/access of centre)<br>▪ Specific study features (free eyeglasses, completeness of eye exam, repair/adjustment of eyeglasses, COMET commitment, association with college of optometry, being part of nationwide study, length of study, selection of frames, eye drops)<br>▪ Incentives/ reinforcements (thank you materials, and newsletters)<br><br>The same scales were used for **staff** surveys, with questions were rephrased to determine how much the staff member believed families liked each survey item.	Percentages of study aspects liked/ disliked, and ratings of importance.<br><br>Significance of differences (chi-squared analysis) between staff and family's rating of importance to retention

Buck et al., 2015	Strabismus	Qualitative arm of pilot RCT - sought to identify parental preferences/ views, on:<br>▪ Study information received<br>▪ Understanding of the need for randomisation<br>▪ Main reasons for participating.<br>▪ Suggestions to enhance acceptability of the future full RCT.	4 Centres (each of which are large centres with specialist paediatric ophthalmology clinics):<br>1) Newcastle upon Tyne National Health Service (NHS) Foundation Trust (coordinating centre)<br>2) Sunderland Eye Infirmary<br>3) Moorfields Eye Hospital, London<br>4) York Hospital NHS Foundation Trust	Parents	▪ 48 (35%) **parents** interviewed from all (n=138) parents of children (age six months – 16 years) eligible for the SamExo Study (29), *including* those who declined SamExo.<br><br>▪ **14** (29%) of the 48 **parents** interviewed, had children who had actually **participated** in the study. NB. The data from non-participating parents was excluded from data extraction and synthesis.<br><br>40 mothers and 8 fathers were interviewed.<br>No data given on the gender, ethnicity or study centre attended for the 14 parents interviewed whose child had participated.	Semi-structured telephone interviews<br><br>Topic guide pre-circulated to parents; interview lasting between 5 and 30 mins; conducted an average of 2 weeks after screening/ consenting (range: 2 days - 10 weeks); not audio recorded; notes taken verbatim by interviewer.<br><br>The interviewer was independent of child's research care – parents were assured of this.	Topic guide relevant questions:<br>▪ Is there anything you think we could change to make the study more acceptable to parents that we will be approaching in the future? - *is there anything else you would like to say about that?*<br>▪ Do you think the Information leaflet explained why randomisation is necessary in this kind of study? *- is there anything else you would like to say about that?*<br>▪ Is there any other information that you would have liked but were not given?<br>▪ Please tell us your main reason for your decision about whether to take part or not.<br>▪ Did you have any other reasons?	Exploratory thematic analysis was conducted by a social scientist/health services researcher. Framework technique was used to develop codes. Inter-rater reliability was conducted by a second researcher coding 50% of the sample.
