# Supplementary material for: Paediatric eye and vision research participation experiences: a systematic review
Source: Trials. 2023 Jan 28;24:66. doi: 10.1186/s13063-022-07021-1 (PMC9883950; doi:10.1186/s13063-022-07021-1)
Supplement: Supplementary file 5 — Additional file 5. Terminology and measures identified to infer ‘experience’ outcomes. [file 13063_2022_7021_MOESM5_ESM.docx]

**Additional File 5:**

**Terminology and measures identified to infer ‘experience’ outcomes**

- Satisfaction’ within a ‘quality of life measure’, for example, the Pediatric Refractive Error Profile (PREP)(Walline et al., 2007).
- ‘Impact of treatment’, measuring the impact or burden of the intervention for the child. For example, the Amblyopia Treatment Index (ATI) (Pediatric Eye Disease Investigator Group Writing Committee, 2010).
- ‘Adverse events’ which collected valuable information about experiences of side effects from interventions. All intervention studies monitor adverse events; from this review we would recommend it could be useful to differentiate between adverse events which are specifically related to the *acceptability* of the intervention.
- ‘Discontinuation narratives’ or ‘reasons for withdrawal’ which were mostly informally presented, but often gave valuable insights of the experience of the intervention. For example, Cho and Cheung (2012) where participants were documented to discontinue owing to failure to comply with treatment.
- ‘Concordance with a test’, ‘success with a test’ or ‘test completion’ which were used for studies evaluating the experience of an assessment. Such terminology often reflected the health professional’s assessment of the test experience. We found that these terms can sometimes be used interchangeably within a study. For example, in Birch et al. (1997) ‘Concordance with a test’ was used in the abstract, then in the full text this was actually measured by a health professional, and rated as a ‘success’ or ‘failure’. Other studies graded an assessment as a ‘success’ if they are ‘completed’.
- ‘Well tolerated’ which we found to have a variety of meanings, relating to ‘compliance’ with a treatment or the ‘acceptability’ of a treatment or relating to ‘adverse events’ as a safety measure, for example.

BIRCH, E., WILLIAMS, C., HUNTER, J. & LAPA, M. C. 1997. Random dot stereoacuity of preschool children. ALSPAC "Children in Focus" Study Team. *J Pediatr Ophthalmol Strabismus,* 34**,** 217-22; quiz 247-8.

CHO, P. & CHEUNG, S.-W. 2012. Retardation of myopia in Orthokeratology (ROMIO) study: a 2-year randomized clinical trial. *Investigative ophthalmology & visual science,* 53**,** 7077-7085.

PEDIATRIC EYE DISEASE INVESTIGATOR GROUP WRITING COMMITTEE 2010. A randomized trial comparing Bangerter filters and patching for the treatment of moderate amblyopia in children. *Ophthalmology,* 117**,** 998-1004. e6.

WALLINE, J. J., GAUME, A., JONES, L. A., RAH, M. J., MANNY, R. E., BERNTSEN, D. A., CHITKARA, M., KIM, A. & QUINN, N. 2007. Benefits of contact lens wear for children and teens. *Eye & contact lens,* 33**,** 317-321.
